# Hemiatrophia Facialis

**Published:** 1890-11-15

**Authors:** 


### HEMIATROPHIA FACIALIS.

Professor Homén reports a case in which carcinoma of the base of the skull had destroyed the N. trigeminus and those of the muscles of the eyeball, leading to anæsthesia of the skin and wasting of the muscles of one side of the face. We have had a case of a girl in whom some central lesion has caused an actual arrest of the development of the entire half of the skull and soft parts, while the other half of the head and face has grown with her years. The atrophic changes have led to complete destruction of the eye on that side. The first symptom of the mischief was rigid contraction of the middle finger on the opposite side, which, however, passed off some years later, before the facial inequality became evident. She is altogether stunted, and one of a highly neurotic family, several members of which are insane.

